# Adapting Mentoring in Times of Crisis: What We Learned from COVID-19

**DOI:** 10.1007/s40596-022-01589-1

**Published:** 2022-02-25

**Authors:** Julie Tetzlaff, Gwen Lomberk, Heather M. Smith, Himanshu Agrawal, Dawn H. Siegel, Jennifer N. Apps

**Affiliations:** Medical College of Wisconsin, Milwaukee, WI USA

Faculty mentoring programs in academic medicine have benefits that range from the individual to the institution and are important to maintain especially during challenging times. Some benefits of faculty mentoring include an enhanced collegial academic culture [1, 2], improved career satisfaction [3–5], increased faculty retention, decreased time to promotion [1, 2, 5, 6], reduced burnout [7], and increased faculty research productivity [1, 3, 4, 8, 9]. Research shows that effective mentors are altruistic in their motivations, exhibit honesty and trustworthiness, and engage in active listening [10]. Mentees who derive maximum benefit earnestly participate in the mentoring relationship, take initiative, have clearly defined objectives, and are open to receiving and incorporating feedback [11].

Throughout the COVID-19 pandemic, both mentors and mentees faced unprecedented uncertainty. As priorities shift and stressors become chronic, the mentoring relationship risks marginalization. However, during these times mentoring needs to remain an important, proactive activity that should not function as crisis management but rather adopt new meaning and adapt to increasing complexities. How does a mentor continue to serve as an effective role model when prior experience from which to draw upon does not exist? Both mentors and mentees have been forced to manage new relational dynamics in an attempt to maintain fidelity to the purpose of the mentoring process, and understanding the psychological responses to stress in both the mentor and mentee can frame how these dynamics may shift in a beneficial manner.

In this commentary, we consider changes within mentoring in academic medicine during the COVID-19 pandemic. We outline several facets of the mentoring process that have been affected by COVID-19, and describe ways mentoring relationships may evolve to respond to these issues. Since the beginning of COVID, several articles have been published offering guidance on how to improve mentoring techniques, such as the importance of maintaining nurturing and consistent mentor-mentee relationships, displaying transparency regarding personal struggles, maintaining active and open communication, acknowledging challenges, and engaging in reflective practices [12–17]. The current paper adds to the literature by expanding on psychological perspectives in order to offer additional suggestions for managing the mentoring relationship during unprecedented times.

## Threats to the Mentoring

Across the globe, people have been surrounded by uncertainty and submerged in anxiety as a result of the pandemic. Individuals react to stress in various ways, with some finding it challenging to adapt to change and others adjusting to uncertainty more easily. The stresses unique to the health care setting during the pandemic have resulted in shifts within the mentor and mentee relationship, and those changes have been complicated by the psychological and behavioral reactions of each individual to increased stress.

Given the global anxiety inherent to a pandemic, one can expect that there might be a temptation and a tendency to discuss more personal details, both from the mentee and the mentor, especially if it is reinforced with positive response from either participant. This can result in significant role diffusion, demonstrated by confusion of previously well-defined social roles between individuals. Professionals in the mental health field could potentially be especially vulnerable to such role diffusions, since interpersonal principles and ethics in psychiatry and psychology can sometimes be unique compared to other medical specialties [18]. For instance, what first may seem like the altruism expected from a strong mentor, interest in the mentee’s management of pandemic-related stress quickly may become a means to gain ideas for one’s own coping. This inadvertent action may turn the mentor away from active listening [19]. Further, virtual meetings may result in sharing more details about one’s personal life than in the past, particularly if those meetings occur while one or more members of the mentoring relationship are at home. In the shift to working from home, it is not uncommon that more families, pets, and even personal décor and items are seen during meetings. For some, these moments may increase a feeling of collegiality, while for others, they may simply feel like poor boundaries. Additionally, more personal discussions may make the mentee less amenable to feedback from the mentor, as feedback also may begin to feel more personal than professional, and the mentee more defensive as a result. We do not mean to suggest that it is always imprudent to discuss more personal details, merely that it is important for the mentor to remain self-aware, self-reflective, and mindful about the purpose and value of self-disclosure. It is crucial for the mentor to continuously examine their motivations for sharing or accommodating more personal details compared to a mentorship session during the pre-COVID era.

Usually, one of the greatest benefits of the mentoring relationship to the mentee is the advanced vantage point mentors have in their professional careers and the resulting experience they share. However, the pandemic has created uncertainty and ambiguity about the future, so our traditional perspectives on career development may be shifting. Both mentor and mentee may find themselves experiencing role reversal, which occurs when individuals acquire each other’s distinct social roles for a time, experiencing and attempting to understand the other person’s perspective. In a time of stress, when role reversal occurs without awareness, it threatens the integrity of the relationship. As mentors are expected to be honest and trustworthy, difficulty acknowledging they lack a certain skill set during the pandemic can threaten this characteristic. For instance, traditionally, the mentee looks at the mentor for guidance and acquisition of skills (e.g., writing, presenting, resolving conflicts, etc.). However, the mentor may find themselves in the pupil’s role as the mentee helps them navigate through the nuances of technology associated with telehealth hardware and software. This can be particularly challenging if there is rigidity to change within the mentor, the mentee, or the system [10]. Additionally, role reversal can detract from a mentee’s initiative for their own goal setting. They may feel disengaged from the process of their own career development as the relationship takes on new dynamics.

## Evolution of the Mentoring Relationship During COVID-19

Given the potential threats to effective mentoring engendered by the pandemic, it is important to consider ways in which the mentoring relationship can change and evolve to ensure continued success (see Table 1).

**Table 1.** Examples of how to implement change in the mentoring relationship during heightened stress

Within the mentoring relationship…	Ways you can make changes:	Things you can say:
Address emotions	• Take time to make a list of things you find stressful and things for which you are grateful • Talk to trusted individuals about your concerns, thoughts, or anxieties around chronic stress • Learn what local resources are available for those at your institution in need of more help	“I am taking time each day to remember why I am grateful for a different work schedule now.” “It is hard for me to admit I don’t have all the answers, and this is a time of a lot of unknowns.” “I learned of a website on our university internal wellness page that can direct you to free therapy or intervention if you think it would be helpful.”
Adjust priorities	• Make an inventory of/audit your current professional tasks and responsibilities • Take time to engage in career planning and goal setting, recognizing it is an ongoing process that will be revisited	“An activity that really helped me was to take time to make a list of all professional tasks I am involved in: teaching, committees, supervision, presentations, manuscripts in progress. Then, given the shifts in my life from the pandemic, I looked at what I should retain, what I could delegate, and what I may need to postpone or decline for now.”
Change formats	• Explore peer mentoring or facilitated peer mentoring rather than (or in addition to) traditional dyad relationships • Experiment with social media such as targeted Facebook groups for professionals • Join listservs for up-to-date information during rapidly changing circumstances	“Now might be a good time to try different mentoring formats, even if you felt in the past they were not a good fit.” “Exploring different mentoring formats is more possible now with virtual options, and only poses minimal risk such as loss of time.” “The more mentoring formats you explore, the better prepared you will be in a variety of situations you could encounter in your career.”
Use technology	• Engage in virtual mentoring options that may not have been possible previously due to time or geographical constraints • Recognize that each person may experience a different level of virtual engagement or fatigue on any given day • Help alleviate the guesswork from virtual relationships by articulating expectations clearly • Do not mistake someone’s challenges (such as with virtual formats) for a lack of expertise in other areas	“This would be a great time to consider joining a peer mentor group with your national professional organization.” “Today is full of back-to-back virtual meetings for me, so it would be really helpful if we plan our time to end ten minutes before the hour, so that we both have a transition moment.” “I will expect everyone to have cameras on during our peer mentor group, so that we can each see our reactions and we can raise hands or use gestures to help the conversation progress more naturally.”
Recognize inequities	• Familiarize yourself with the underrepresented groups in your area of practice and the programming available at your institution • Recognize that you may be unaware of challenges or limitations in the lives of others • Explore your own comfort, or lack thereof, with discussing topics related to inequity and be ready to articulate boundaries appropriate for yourself	“I want you to know that I care about your needs and how those might influence your career. Have things been more difficult to manage than normal during the pandemic?” “Help me understand what the limitations are that influence your ability to progress in your career.” “I admit that I do not fully understand or know what to do about these problems. Perhaps we should look into what our institution’s Center on Diversity and Inclusion offers?”
Remember the basics	• Engage in active listening • Avoid multi-tasking while engaging in a virtual format • Remember that effective feedback should assume positive intent, be objective, concise and direct but open and conversational • Remember that we all struggle to accept change	“I am going to close my Inbox during this meeting, so I am not distracted by my incoming email.” “I want to give you feedback about the draft you submitted. You incorporated our prior feedback well, but the last section is still disorganized. I realize you have other duties given to you lately and time is short. Can you talk to me about your plan to address the last section?” “I still feel disappointed in myself some days when I don’t get more accomplished. It is hard to remember that I am juggling many changes in how I work and that impacts my productivity some days.”

### Address Emotions

Recognize your own comfort with vulnerability in order to address and deal with emotions during this time of stress. As humans, we all have vulnerabilities, although these may have been hidden behind boundaries in the past within the mentoring relationship. Mental health professionals have training in recognition and management of emotions, which could imply they should be more efficient at recognizing their and other’s vulnerabilities. However, simultaneously some mental health professionals may struggle with knowing how to express their emotions overtly without violating what in the past have been held as clear limits. The experience of chronic, universal stress requires us each to consider our level of comfort with vulnerability. Allow space to process emotions, and this will enhance the honesty and trust within the mentoring relationship. Vulnerability is strength, and most of all, understanding one’s own level of comfort, awareness, and expression of vulnerability is strength. Flexibility and adaptability are essential as mentoring relationships allow for consideration of unexpected topics of conversation and processing of emotional reactions to the current state. However, it remains incumbent upon mentors to monitor their level of vulnerability and not impart this too heavily on mentees. All of us should be familiar with how to complete a basic “risk assessment” with our mentees, and how to suggest or encourage additional intervention if someone appears to be struggling. Being aware of your own comfort with emotional content also can inform an approach to asking about your mentee’s emotional health. In times of significant stress, recognizing signs of poor coping or mental health needs becomes even more important. Take time to learn local available resources for more formal intervention and share these with your mentees.

### Adjust Priorities

Respond to issues as they arise, recognizing that this may temporarily change mentoring priorities. Mentoring relationships that are built around a specific goal may feel strained during the pandemic as one or both parties have a shift of priorities or experience feelings of futility around their original goal. Be ready to adjust priorities as needed. Spend time completing new goal setting exercises that do not lose the mentee’s ultimate long-term aspirations but modify short- and mid-term goals to accommodate new problems. Maintain respect for the mentor’s experience and reputation within the mentoring relationship, even if the focus shifts around one topic temporarily. For example, even though certain biological, psychological, and social circumstances of the world may have changed for a mentee during a pandemic, the mentor may still be the relative authority on the bio-psycho-social model.

### Change Formats

Explore role reversal openly or consider other forms of mentoring relationships for a time if necessary. Unprecedented and uncertain times may be the ideal opportunity to explore different types of mentoring paradigms. Several distinct styles of mentoring have been reviewed in the literature [20]. Historically, dyad relationships are most common, and mental health professionals who practice psychotherapy (including psychiatrists, psychologists, social workers, etc.) may have an advantage owing to their familiarity with this model. However, this model can sometimes be limited by its hierarchical structure and restricted range of advice available from a single mentor. Increasingly common are peer and facilitated peer mentoring groups. Within the peer mentoring paradigm, individuals of similar academic rank and experience mentor each other. This method can be limiting due to the lack of collective experience among group members. To circumvent this, consider participating in facilitated peer mentoring, which recruits a senior leader to mentor the peer group. Alternatively, or in addition to, a mentee might explore more passive yet informative methods of peer mentoring such as those provided by social media. Cree-Green(2020) [21] describes members-only interactive groups on Facebook as a method to more passively engage with peers. Another example could include joining a targeted listserv, a means of connecting with peers which can be a valuable mechanism to share rapidly evolving information in a pandemic situation.

### Utilize Technology

Use technology effectively while also acknowledging the fatigue and isolation resulting from a transition to a predominantly virtual life. Virtual meetings have allowed a mechanism for continued mentoring in the midst of the pandemic, although many feel a sense of lost human connection due to the limited ability for face-to-face, in-person encounters. The use of videoconferencing in the mentoring relationship has several advantages, as well as limitations. It allows interactions and opportunities for mentorship between individuals in geographically distanced locations that otherwise might not have been possible. It eliminates travel to allow for meetings to take place in a narrower window of time. However, the loss of in-person contact and limitations with nonverbal communication challenge the mentor-mentee dynamic. Exclusively virtual communication may lead to lower satisfaction and poorer perceptions of the interaction [22]. Decreased need for travel also has resulted in increased demands on time, as the previously required transition time between meetings is eliminated. This results in virtual fatigue, and a mentor must remain mindful of this and discuss with mentees on the importance of new versions of boundary setting, time management, and personal scheduling. Additionally, as mentioned before, videoconferencing can promote a loss of anonymity. Now mentors and mentees may get a view of one another’s homes or family life. While in some cases this may lead to a closer relationship, in others it may cross a professional boundary [23]. It remains important for a mentor to be aware of their own level of virtual engagement or fatigue, while also gauging the status of the mentee. Recognize that when all meetings have the same virtual appearance, it can be challenging for a mentee to understand expectations for their behavior and for the interaction. Make a point, as the mentor, to announce intentions for the relationship, and be upfront about articulating expectations.

### Recognize Inequities

Recognize that pre-existing societal burdens and inequities have become exacerbated during this time. Historically, underrepresented groups and women have struggled with various aspects of faculty development, including but not limited to a sense of family and/or community obligations, feelings of isolation, and lack of suitable mentors [24–27]. During the COVID-19 pandemic, many of these stressors likely not only extend to additional groups within the academic environment in a universal manner but are uniquely intensified for those confronting them pre-pandemic. In addition, we have entered a scenario in which the mentors are encountering their own increased stressors. Ensuring that the mentoring relationship is safe to discuss these experiences will continue to be important. Recognize that both mentors and mentees may be engaging in roles novel to them, or even new to conceptualization.

### Remember Basics

Remember the basics of mentoring. Success in mentoring involves active engagement and initiative. Both parties must actively listen to one another, with a willingness to incorporate feedback. Historically, mentoring success often revolved around initiative rather than reactivity. However, it becomes difficult to think ahead in a situation requiring constant change and reaction. Intense stress can lead to defensiveness and challenges the ability to remain in the moment and focused, let alone open-minded. Acknowledge that we are all finding a new process of moving forward. We need to discover ways to remain connected to our compassion and to set new expectations for what is possible right now [28]. As mentors, we can recognize that mentees in more vulnerable or early stages of career development may benefit from more frequent, basic touch points [29]. Recognize that chronic stress will leave everyone more fatigued on all levels, and that means we may not engage in the same frequency or types of mentoring as we have in the past. Productivity can look different during this time.

## Conclusions

As with any time of increased stress, the strain of a global pandemic requires those in academic medicine to consider their coping strategies and resiliency. Within the mentor and mentee relationship, dynamics have shifted as a result of COVID-19. We encourage academic medicine faculty to remember the importance of mentoring during these times and to focus on adjusting expectations and approach in order to accommodate increased pressure, excess demands, and emotional responses. By utilizing the mentoring relationship as an opportunity to increase our emotional awareness and practice increased patience and grace with ourselves and others, we create an environment that allows both the mentor and the mentee to evolve through crisis and to integrate the benefits of the mentoring relationship as permanent changes that transcend well beyond COVID-19.
